# Supplementary material for: Effects of exercise intervention on executive function in children with overweight and obesity: a systematic review and meta-analysis
Source: PeerJ. 2025 Apr 14;13:e19273. doi: 10.7717/peerj.19273 (PMC12005193; doi:10.7717/peerj.19273)
Supplement: Supplemental Information 5 [file peerj-13-19273-s005.docx]

**Search strategy：**

| PubMed Search strategy | | |
| --- | --- | --- |
| #1 | Exercise[MeSH Terms] | 261414 |
| #2 | (((((((((((((((((((((((((((((((((((((((((((((((((((((((aerobic exercise[Title/Abstract]) OR (aerobic training[Title/Abstract])) OR (Physical Activity[Title/Abstract])) OR (Physical Exercise[Title/Abstract])) OR (Taijiquan[Title/Abstract])) OR (Dancing[Title/Abstract])) OR (Cycling[Title/Abstract])) OR (Swimming[Title/Abstract])) OR (Jogging[Title/Abstract])) OR (walking[Title/Abstract])) OR (vigorous walking[Title/Abstract])) OR (treadmill[Title/Abstract])) OR (qi gong[Title/Abstract])) OR (ba dual jin[Title/Abstract])) OR (stair climbing[Title/Abstract])) OR (isometric[Title/Abstract])) OR (acute exercise[Title/Abstract])) OR (endurance training[Title/Abstract])) OR (sports[Title/Abstract])) OR (athletics[Title/Abstract])) OR (resistance training[Title/Abstract])) OR (strength training[Title/Abstract])) OR (resistance exercise[Title/Abstract])) OR (strength exercise[Title/Abstract])) OR (weight training[Title/Abstract])) OR (weight-lifting strengthening program[Title/Abstract])) OR (weight lifting exercise program[Title/Abstract])) OR (high-intensity interval training[Title/Abstract])) OR (high-intensity intermittent exercise[Title/Abstract])) OR (sprint interval training[Title/Abstract])) OR (hint[Title/Abstract])) OR (hint[Title/Abstract])) OR (circuit-based exercise[Title/Abstract])) OR (plyometric exercise[Title/Abstract])) OR (plyometric training[Title/Abstract])) OR (stretch shortening cycle exercise[Title/Abstract])) OR (stretch-shortening exercise[Title/Abstract])) OR (plyometric drills[Title/Abstract])) OR (cycle exercise[Title/Abstract])) OR (ball[Title/Abstract])) OR (soccer ball[Title/Abstract])) OR (soccer[Title/Abstract])) OR (football[Title/Abstract])) OR (basketball[Title/Abstract])) OR (ping-pong[Title/Abstract])) OR (badminton[Title/Abstract])) OR (tennis[Title/Abstract])) OR (baseball[Title/Abstract])) OR (volleyball[Title/Abstract])) OR (softball[Title/Abstract])) OR (racquet sport[Title/Abstract])) OR (racket sport[Title/Abstract])) OR (lacrosse[Title/Abstract])) OR (racquetball[Title/Abstract])) OR (exercise[Title/Abstract]) | 807940 |
| #3 | #1 OR #2 | 856905 |
| #4 | executive function[MeSH Terms] | 20984 |
| #5 | (((((((((((((((executive function[Title/Abstract]) OR (executive control[Title/Abstract])) OR (cognitive function[Title/Abstract])) OR (cognitive performance[Title/Abstract])) OR (inhibitory control[Title/Abstract])) OR (shifting[Title/Abstract])) OR (working memory[Title/Abstract])) OR (refresh function[Title/Abstract])) OR (cognitive flexibility[Title/Abstract])) OR (planning[Title/Abstract])) OR (cognitive function[Title/Abstract])) OR (reasoning[Title/Abstract])) OR (problem solving[Title/Abstract])) OR (updating[Title/Abstract])) OR (inhibition[Title/Abstract])) OR (attention[Title/Abstract]) | 2146509 |
| #6 | #4 OR #5 | 2150993 |
| #7 | (((Overweight[Title/Abstract]) OR (Obesity[Title/Abstract])) OR (Obese[Title/Abstract])) OR (excess weight[Title/Abstract]) | 436091 |
| #8 | Child[MeSH Terms] | 2224865 |
| #9 | (Child[Title/Abstract]) OR (Children[Title/Abstract]) | 1588649 |
| #10 | #8 OR #9 | 2721065 |
| #11 | #3 AND #6 AND #7 AND #10 | 852 |

| Web of Science Search strategy | | |
| --- | --- | --- |
| #1 | (TS=(Executive Function) OR AB=(Executive Control OR Cognitive Function OR cognitive performance OR inhibitory control OR Shifting OR working memory OR Refresh function OR cognitive flexibility OR Planning OR cognitive function OR Reasoning OR problem solving OR Updating OR inhibi-tion OR attention)) | 4695670 |
| #2 | (TS=(Child) OR AB=(Children)) | 2140856 |
| #3 | (TS=(Overweight,Obesity) OR AB=(Obese OR excess weight OR Overweight OR Obesity)) | 385408 |
| #4 | (TS=(Exercise) OR AB=(aerobic exercise OR aerobic training OR Physical Activity OR Physical Exercise OR Taijiquan OR Dancing OR Cycling OR Swimming OR Jogging OR walking OR vigorous walking OR Treadmill OR qi gong OR ba dual jin OR stair climbing OR Isometric OR Acute Exercise OR Endurance Training OR Sports OR Athletics OR Resistance Training OR Strength Training OR Resistance Exercise OR Strength Exercise OR weight training OR Weight-Lifting Strengthening Program OR Weight Lifting Exercise Program OR high-intensity interval training OR High-Intensity Intermittent Exercise OR Sprint Interval Training OR hint OR yoga OR Circuit-Based Exercise OR Plyometric Exercise OR Plyometric Training OR Stretch Shortening Cycle Exercise OR Stretch-Shortening Exercise OR Plyometric Drills OR Cycle Exercise OR ball OR soccer ball OR Soccer OR Football OR basketball OR ping-pong OR badminton OR Tennis OR Baseball OR Volleyball OR Softball OR Racquet Sport OR Racket Sport OR Lacrosse OR Racquetball OR exercise OR dancing)) | 5812566 |
| #5 | #1 AND #2 AND #3 AND #4 | 1375 |
