# Supplementary material for: Effects of exercise intervention on executive function in children with overweight and obesity: a systematic review and meta-analysis
Source: PeerJ. 2025 Apr 14;13:e19273. doi: 10.7717/peerj.19273 (PMC12005193; doi:10.7717/peerj.19273)
Supplement: Supplemental Information 1 [file peerj-13-19273-s001.docx]

Dear Editor,

The strength of this study is the integration of multiple disciplines, including kinesiology, psychology, and obesity. Most previous studies have explored the impact of exercise intervention on the executive function of children with normal weight, while this study explores the impact of exercise intervention on the executive function of children with overweight and obesity, which is innovative in the object of study.

In addition, this study also explored in detail the moderating variables affecting the impact of exercise interventions on the inhibitory control of children with overweight and obesity (exercise duration, exercise intensity, exercise type, and age). This is also an innovative aspect of this research.

We believe that readers in the fields of kinesiology, psychology and medicine will enjoy this study and provide them with some reference value.
